# FACTORS ASSOCIATED WITH DEPRESSIVE SYMPTOMS AMONG OLDER SOUTHEAST ASIAN AMERICANS

**DOI:** 10.1093/geroni/igad104.0484

**Published:** 2023-12-21

**Authors:** Kaipeng Wang, My Ngoc To, Carson De Fries, Bei Wu

**Affiliations:** University of Denver, Denver, Colorado, United States; University of Denver, Denver, Colorado, United States; University of Denver, Denver, Colorado, United States; New York University, New York City, New York, United States

## Abstract

Depressive symptoms are prevalent among older adults, particularly those from Southeast Asian American (SEAA) communities. Although SEAAs represent one of the fastest growing minority groups in the U.S, research has given little attention to understanding depression among older SEAAs. This study therefore aimed to examine the factors associated with depressive symptoms among older Southeast Asian Americans. We recruited a total of 251 adults aged 55 and above who identified as Vietnamese American, Filipino American, or Indonesian American via community organizations. Participants completed online or in-person questionnaires. Depressive symptoms were measured with the 10-item Center for Epidemiologic Studies Depression Scale. Items are rated on a 4-point Likert scale, with higher scores indicating more depressive symptoms. We used ordinary least squares (OLS) regression to examine the association between depressive symptoms and various health, psychological, social, demographic, and environmental factors. In general, Vietnamese Americans (mean=1.74, SD=0.65) had significantly more depressive symptoms than Indonesian (mean=1.56, SD=0.60) and Filipino (mean=1.47, SD=0.47) Americans. OLS regression results showed that resilience and age were negatively associated with depressive symptoms, while greater family conflict was significantly associated with more depressive symptoms. Participants who could not speak English fluently had significantly more depressive symptoms than those who could not. Those residing in urban or suburban areas had significantly more depressive symptoms than those living in rural areas. Results contribute to better understanding of the factors that contribute to depression among older SEAAs and inform the strategies for preventing and treating depression in this population.

